# Regional Variations in and Key Predictors of Feline Tumor Malignancy: A Decade-Long Retrospective Study in Korea

**DOI:** 10.3390/ani14202989

**Published:** 2024-10-16

**Authors:** Byung-Joon Seung, Min-Kyung Bae, Jung-Hyang Sur

**Affiliations:** 1 Department of Veterinary Pathology, College of Veterinary Medicine, Konkuk University, Seoul 05029, Republic of Korea; mkbae4136@gccorp.com; 2 Department of Pathobiology, College of Veterinary Medicine, University of Illinois at Urbana-Champaign, Urbana, IL 61802, USA; 3 Research Institute, Green Vet, Yongin-si 16907, Republic of Korea; 4 Komipharm International Co., Ltd., Siheung-si 15094, Republic of Korea

**Keywords:** feline tumors, malignancy, age, Korean cats, retrospective study

## Abstract

Feline cancer is increasingly recognized as a major cause of mortality, yet data on tumor prevalence and behavior in cats, particularly in non-Western regions, remain limited. This study analyzed a decade of feline tumor data in Korea from 2012 to 2022, focusing on age, breed, and anatomical location as predictors of malignancy. Data were collected from 683 cats, with regression analysis applied to determine significant associations. Older cats exhibited a markedly higher risk of malignancy, particularly in mast cell and mammary tumors. Tumors in the mammary gland and alimentary tract had malignancy rates exceeding 90%, underscoring the need for early detection in these regions. Interestingly, squamous cell carcinoma was rare in the skin, in stark contrast to Western studies, likely reflecting differences in environmental exposure. While breed was not a statistically significant predictor, certain breeds, including Persians and Russian Blues, showed a higher frequency of malignancy. These findings highlight the importance of regional tumor research in cats and the need for larger, multicenter datasets that incorporate environmental, genetic, and lifestyle factors. Understanding these influences will help refine veterinary care and improve cancer treatment outcomes in feline populations.

## Simple Summary

Cancer is becoming a bigger health issue for cats, much like it is for humans. However, most research has focused on dogs, leaving a gap in our understanding of how cancer affects cats, especially in countries like Korea. In this study, we looked at ten years of data on cats diagnosed with tumors in Korea, aiming to find out which factors—like age, breed, or where the tumor is located—might increase the risk of the cancer being serious or malignant. We discovered that older cats were much more likely to have aggressive cancers, especially in areas like the mammary glands and digestive system. On the other hand, some types of cancer, like squamous cell carcinoma (a type of skin cancer), were much less common in Korea than in other countries. These findings can help veterinarians better understand which cats are at higher risk and encourage early diagnosis and treatment. This research adds to what we know about feline cancer and helps improve the care we provide for cats around the world.

## 1. Introduction

Cancer is a leading cause of mortality in companion animals and has become a growing concern, particularly in feline populations [1,2]. Although the incidence of tumors in cats is generally considered lower than in dogs [1,2], recent evidence suggests that cancer is emerging as one of the primary causes of death in cats [3,4].

Cancers in companion animals share significant similarities with human cancers, especially in terms of their natural disease progression [5]. Both canine and feline cancers hold valuable potential as models for comparative oncology research, given cats and dogs have similar environmental exposures to those of humans [6,7]. While dogs have been extensively studied as comparative oncology models, resulting in a large body of tumor data, research in cats has been more limited, leading to a comparatively smaller dataset and fewer insights into feline oncology [4,8].

In South Korea, cats are now the second most owned pet after dogs [9], with a sharp rise in the number of pet-owning households in recent years. However, most studies on feline tumor prevalence have focused on Western populations, particularly in the United States and Europe [10,11,12,13,14,15,16]. Due to ecological and environmental differences between regions, findings from these studies may not be directly applicable to Asian feline populations. For example, the Swiss Feline Tumor Registry [10], which includes data from 51,322 feline patients between 1965 and 2008, serves as a global point of reference. While some studies have been conducted in Asia, such as those in Korean and Japan [17,18], the available data are significantly more limited compared to Western countries, emphasizing the need for more region-specific research in Asian populations.

Given this context, epidemiological data on feline tumors—particularly regarding factors such as breed, sex, and age—are essential for enhancing our understanding of tumor prevalence and distribution in feline populations. This information can assist veterinarians in recognizing patterns and potential risk factors associated with different tumor types. In the present study, we analyzed the prevalence of feline tumors in Korea from 2012 to 2022, investigating the relationship between major tumor types and demographic factors, including breed, sex, and age.

## 2. Materials and Methods

This retrospective study analyzed feline tumor histopathology records archived between 2012 and 2022 at the Department of Veterinary Pathology, Konkuk University, Seoul, South Korea. The tissue samples were collected with the consent of the owners as part of routine diagnostic and treatment procedures at various primary care and specialty veterinary hospitals for privately owned cats. No samples were collected specifically for research purposes and, as such, ethical approval was not required. This study relied solely on previously collected diagnostic records.

Cats were included in the study based on two criteria: (1) a confirmed histopathological diagnosis of at least one tumor and (2) the availability of complete histopathology records regarding breed, age, and sex. To ensure accurate epidemiological data, each distinct histological tumor type identified in a single cat was recorded as a separate case. Conversely, when multiple occurrences of the same tumor type were found in the same cat, these were collectively treated as a single case to avoid overrepresentation of that tumor type.

Tumor diagnoses were made following standard histopathological procedures. After surgical excision, tissue samples were fixed in 10% neutral-buffered formalin and processed for routine histological examination. Sections, 4 µm thick, were cut from formalin-fixed, paraffin-embedded (FFPE) blocks and stained with hematoxylin and eosin (H&E) for evaluation. Tumors were classified and diagnosed according to the latest nomenclature [19]. In cases where classification proved challenging, immunohistochemistry (IHC) was performed for further diagnostic clarity. Cases without IHC, where the diagnosis remained inconclusive, were excluded from this study.

Following initial classification, tumors were categorized as either benign or malignant. For certain tumor types, additional classification criteria were applied. For instance, mast cell tumors (MCTs), although they generally behave benignly, may carry a poor prognosis under specific conditions, such as the following: (1) mitotic count >5/10HPF, (2) the presence of five or more tumors, or (3) evidence of metastasis, including to nearby lymph nodes [20]. When any of these conditions were met, the tumor was classified as malignant. Malignant mammary tumors were also graded when possible, using a recently proposed grading system that incorporates lymphovascular invasion, nuclear morphology, and mitotic count [21]. For peripheral nerve sheath tumors, all were classified as malignant, in line with the most recent edition, which does not distinguish between benign and malignant forms [22].

Data collected for analysis included age, sex, breed, tumor type, malignancy status, and histological grade. Descriptive analyses were performed using counts, medians, and percentages for categorical variables and means with standard deviations (SDs) for continuous variables. To assess the difference in the average age at diagnosis between benign and malignant tumors, an unpaired *t*-test was performed. Age was grouped into quartiles as follows: quartile 1 (Q1), <6 years; quartile 2 (Q2), 6–8 years; quartile 3 (Q3), 9–11 years; and quartile 4 (Q4), >11 years. Data were analyzed using chi-square or Fisher’s exact tests (employing Monte Carlo simulation), with significant variables further examined through multiple logistic regression models. A *p*-value of <0.05 was considered statistically significant, and odds ratios (ORs) with 95% confidence intervals (CIs) were calculated. Statistical analyses were performed using IBM SPSS Statistics for Windows, Version 27.0.

## 3. Results

This retrospective study initially reviewed records from 892 cats undergoing tissue evaluations, from which 683 cats were selected for the analysis. This included a total of 689 tumors, as some cats presented with multiple tumors. Tumors of the same histological type occurring at multiple sites were counted as a single case to avoid overrepresentation, while distinct histological tumor types in the same cat were recorded as separate cases. This approach ensures that our statistics reflect the true diversity of tumors within the population and the individual burden of each tumor type. Among these tumors, 316 (45.9%) were classified as benign, while 373 (54.1%) were classified as malignant. All sample characteristics are presented in Supplementary Table S1.

### 3.1. Age

**Figure 1:**
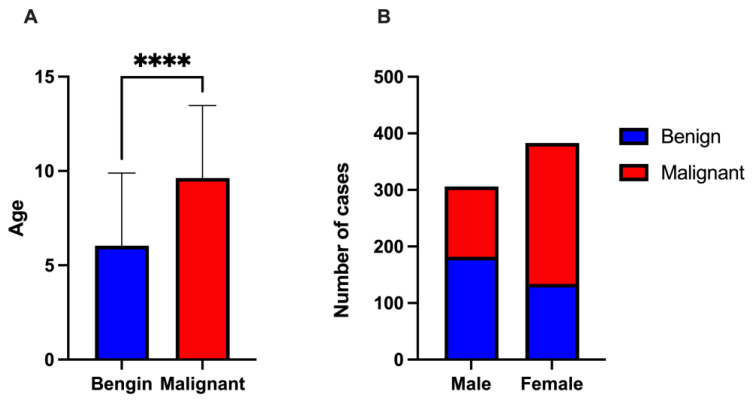
Distribution of benign and malignant tumors in cats. (**A**) Distribution by age, with error bars representing standard deviations (SDs) to show variability. Asterisks (****) indicate statistically significant differences between groups (*p* < 0.0001). (**B**) Distribution by sex, illustrating differences in tumor prevalence between males and females.

The cats in the dataset ranged from 0 to 20 years old, with an average age of 8.08 ± 3.95 years. Benign tumors were diagnosed at a younger average age of 6.60 ± 3.81 years, while malignant tumors occurred at an older average age of 9.33 ± 3.62 years (*p* < 0.0001, Figure 1A). The most common age was 8 years, representing 11.3% of the total cases (78 cats). Female cats tended to be older, with an average age of 8.60 ± 3.93 years compared to 7.42 ± 3.88 years for males. An unpaired *t*-test revealed a significant association between age and tumor malignancy (*p* < 0.0001). Additionally, a chi-square test revealed a significant association between age quartiles and tumor malignancy (*p* < 0.001).

### 3.2. Sex

Of the 689 tumors, 383 (55.6%) occurred in female cats, while 306 (44.4%) were found in males. Among the female cats, 134 tumors (35.0%) were benign and 249 (65.0%) were malignant. In contrast, 182 tumors (59.5%) in males were benign, with 124 (40.5%) classified as malignant. Malignant tumors were more prevalent in female cats, whereas benign tumors were more common in males (Figure 1B). A chi-square test revealed a significant association between sex and tumor malignancy (*p* < 0.001).

### 3.3. Breed

**Figure 2:**
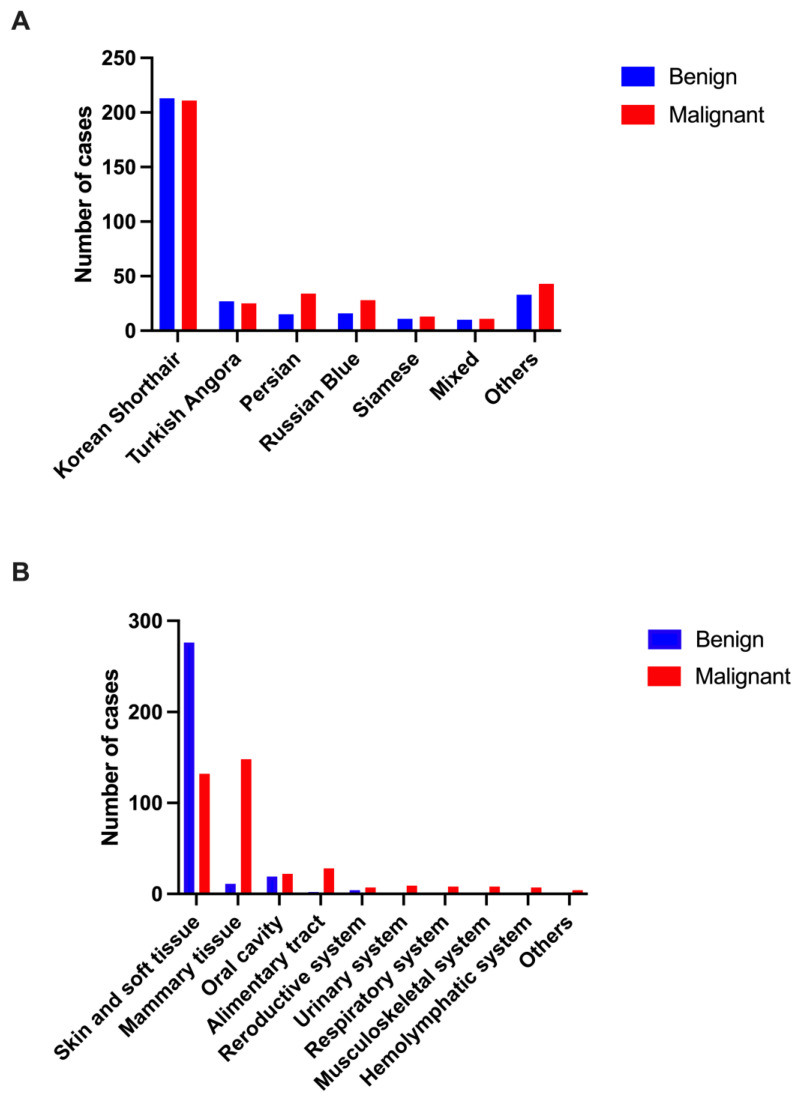
Patterns of tumor distribution by breed and anatomical location in cats. (**A**) Distribution by breed, highlighting the comparison of benign and malignant tumor cases among various cat breeds. (**B**) Distribution by anatomical location, illustrating the prevalence of tumor types in different body regions.

The Korean Shorthair, the most common breed in Korea, accounted for the largest proportion of cases in this study, representing 61.3% of the total (424 cats). These cats exhibited an almost equal distribution of benign and malignant tumors, with 213 benign (49.2%) and 211 malignant (50.8%) cases. The Turkish Angora was the second most prevalent breed, with an equal distribution of 26 benign (50.0%) and 26 malignant (50.0%) tumors. Persian cats showed a higher propensity for malignancy, with 34 malignant tumors (69.4%) and 15 benign tumors (30.6%) out of 49 cases. Similarly, Russian Blue cats presented with 28 malignant tumors (63.6%) out of 37 cases. The Siamese breed had 24 cases, 11 benign (45.8%) and 13 malignant (54.2%). In the Mixed group, comprising 21 cats, 12 had benign tumors (57.1%) and 9 had malignant tumors (42.9%). Breeds categorized as ‘Others’ included British Shorthair, American Shorthair, Bengal, Scottish Fold, Norwegian Forest, Abyssinian, and other rare breeds. These breeds were less represented in this study, with fewer cases and varying distributions of benign and malignant tumors. The chi-square test did not reveal a significant association between breed and tumor malignancy (*p* = 0.173). Figure 2A presents a visual comparison of the distribution of benign and malignant tumors across different breeds, illustrating the number of malignant cases by breed.

### 3.4. Anatomic Location

Tumors were distributed across various anatomical locations, with notable differences in the proportion of benign and malignant cases. Of the 689 tumors, 408 (59.2%) were in the skin and subcutis, where 276 (67.6%) were benign and 132 (32.4%) were malignant. The mammary tissue accounted for 159 tumors, the vast majority of which were malignant (148 cases, 93.1%). Tumors in the alimentary tract also displayed a predominantly malignant profile, with 28 out of 30 cases (93.3%) classified as malignant and only 2 (6.7%) as benign.

Other regions, such as the reproductive system (11 cases) and urinary system (10 cases), followed a similar trend. In the reproductive system, seven tumors (63.6%) were malignant and four (36.4%) were benign, while in the urinary system, nine tumors (90%) were malignant and one (10%) was benign. Malignant tumors were particularly common in the respiratory system (88.9%, eight out of nine cases), in the musculoskeletal system (100%, eight out of eight cases), and in the hemolymphatic system (87.5%, seven out of eight cases), emphasizing the aggressive nature of tumors in these regions. Fisher’s exact test, performed using Monte Carlo simulation, demonstrated a statistically significant association between tumor location and malignancy (*p* < 0.001), indicating that certain anatomical sites, such as the mammary tissue and alimentary tract, were more prone to malignancies.

Table 1 and Figure 2B provide a detailed breakdown of these proportions, visually illustrating the distribution of benign and malignant tumors across all anatomical locations.

#### 3.4.1. Skin and Soft Tissue

The skin and soft tissue were the most common sites for tumors, with 408 cases. Among these, MCTs were the most frequently diagnosed, comprising 172 benign cases and 43 malignant cases. Benign MCTs were diagnosed at a younger average age of 5.64 ± 3.56 years (range: 0 to 16 years), whereas malignant MCTs typically occurred later in life, with an average age of 8.90 ± 3.69 years. Male cats were more frequently affected by both benign (61.9%) and malignant (57.1%) MCTs.

A chi-square test was performed to assess the relationship between the benign/malignant status of MCTs and various factors, including age quartiles. The results demonstrated a statistically significant association between age quartile and tumor malignancy (*p* < 0.001). Most benign tumors were found in younger cats, particularly in the Q1 age group (<6 years), where 92.5% of tumors were benign. As age increased, the proportion of malignant tumors also rose, with 41.9% of tumors in the Q3 age group (9–11 years) and 38.5% in the Q4 age group (>11 years) being malignant.

Other benign tumors included lipoma (n = 51), which primarily affected male cats (52.9%) with a mean age of 8.16 ± 2.78 years. Additionally, fibroma (n = 10) was recorded among the benign cases. Among the malignant tumors, fibrosarcoma (n = 35) was notable, with a higher prevalence in females (68.6%) and a mean age of 7.51 ± 3.11 years. Melanocytic tumors included melanocytomas (n = 2) and amelanotic melanomas (n = 4), the latter being particularly notable for their lack of pigmentation (a factor that is associated with poorer prognosis [23]). Less common malignant tumors included hemangiosarcoma (n = 3) and squamous cell carcinoma (SCC) (n = 3), which generally occurred in older cats.

#### 3.4.2. Mammary Tissue

Mammary tissue accounted for 159 tumors, of which 92.3% (n = 148) were malignant. Mammary carcinoma was the most common diagnosis, occurring exclusively in female cats. The mean age of cats with mammary carcinoma was 10.29 ± 3.01 years, with ages ranging from 2 to 20 years. Among the malignant cases, mammary carcinomas were classified into three grades: high-grade (n = 77, 53.8%), intermediate-grade (n = 25, 17.5%), and low-grade (n = 21, 14.7%).

In contrast, 11 benign mammary adenomas were recorded, with a mean age of 5.55 ± 4.61 years, ranging from 1 to 13 years. A chi-square test was performed to assess the relationship between tumor malignancy (benign or malignant) and age quartiles in cats with mammary tumors. The results revealed a statistically significant association between age quartile and malignancy status (*p* < 0.001).

Malignant tumors were more frequently observed in older cats, particularly in the Q3 age group (9–11 years), where 98.4% of tumors were malignant, and in the Q4 age group (>11 years), where 96.2% were malignant. In contrast, benign tumors were more common in younger cats: in the Q1 age group (<6 years), 41.2% of tumors were benign, while in the Q2 age group (6–8 years), only 1 benign tumor (3.7%) was recorded, with the remainder being malignant.

Although age was significantly associated with tumor malignancy, the chi-square analysis showed no statistically significant association between tumor grade and age quartiles (*p* = 0.208). These findings suggest that while age is a strong factor influencing malignancy, it does not significantly affect the grade of malignant tumors.

#### 3.4.3. Oral Cavity

In the oral cavity (n = 41), peripheral odontogenic fibromas (n = 16) were the most common benign tumors, with a mean age of 6.31 ± 3.00 years, equally affecting males (50.0%) and females (50.0%). SCC (n = 16), the most common malignant tumor, had a mean age of 9.31 ± 3.55 years, with 37.5% occurring in males and 62.5% in females. Additionally, rare cases of fibrosarcoma (n = 4) and salivary adenocarcinoma (n = 2) were observed, typically in older cats.

#### 3.4.4. Alimentary Tract

The alimentary tract was a common site for malignancy, with a total of 30 cases, 93.3% (n = 28) of which were malignant. Lymphoma was the most frequent diagnosis, accounting for 22 cases. It is important to note that while lymphomas, particularly the more frequently occurring types such as small cell lymphomas, are generally noted for their slower progression [24], our study did not specifically classify lymphomas by cell type. The mean age of cats diagnosed with lymphoma was 8.41 ± 4.14 years, and it was more prevalent in males (68.2%). Other malignant tumors included intestinal adenocarcinomas (n = 3) and a gastrointestinal stromal tumor (n = 1). Benign tumors in the alimentary tract were rare, with only two cases recorded: one gastric adenoma and one leiomyoma.

#### 3.4.5. Reproductive System

The reproductive system had 11 cases, the majority of which were malignant. Dysgerminomas (n = 2) and uterine adenocarcinomas (n = 2) were exclusively malignant, occurring in female cats with mean ages of 9.00 ± 4.24 years and 8.00 ± 1.41 years, respectively. Leiomyosarcomas (n = 2) were also malignant, with a mean age of 4.50 ± 2.12 years. In contrast, benign tumors were less common. These included a lipoma (n = 1), luteoma (n = 1), uterine adenoma (n = 1), interstitial cell tumor (n = 1), and granulosa cell tumor (n = 1), with ages ranging from 0 to 14 years.

#### 3.4.6. Urinary System

The urinary system had 10 cases, with renal cell carcinoma (n = 6) being the most common, primarily affecting males (66.7%) with a mean age of 11.00 ± 2.10 years. Other diagnoses included urothelial carcinoma (n = 3) and leiomyoma (n = 1).

#### 3.4.7. Respiratory System

All nine tumors in the respiratory system were malignant. These included lymphomas (n = 3), nasal carcinomas (n = 3), and pulmonary adenocarcinomas (n = 2). The mean age for nasal carcinoma was 6.33 ± 2.08 years, with an equal distribution between males and females.

#### 3.4.8. Musculoskeletal System

All eight tumors in the musculoskeletal system were malignant. Osteosarcoma (n = 5) was the most common, with a mean age of 8.00 ± 3.46 years. Other malignant tumors included chondrosarcoma (n = 2) and rhabdomyosarcoma (n = 1), which were observed in older cats.

#### 3.4.9. Hemolymphatic System

There were eight tumors in the hemolymphatic system, with lymphoma being the most frequent diagnosis (n = 4), equally distributed between males and females. Other tumors included extramedullary plasmacytoma (n = 2) and fibrosarcoma (n = 1).

#### 3.4.10. Other Sites

Lastly, five cases were classified under “other” anatomical sites. These included rare diagnoses such as hepatocellular carcinoma (n = 2) and mesothelioma (n = 1).

### 3.5. Multiple Logistic Regression Analysis

To further evaluate the independent effects of tumor location, age, and sex on the likelihood of malignancy, a multiple logistic regression analysis was conducted. The results of this analysis are summarized in Table 2.

In the assessment of tumor location as a predictor of malignancy, the alimentary tract displayed the highest odds of malignancy (OR = 34.927, 95% CI: 7.998–152.521, *p* < 0.001). Notably, this elevated risk is predominantly associated with lymphomas, especially the frequently occurring types known for their slower progression, though our study did not classify lymphomas by specific cell types. Tumors in the urinary system also had significantly higher odds of malignancy (OR = 17.234, 95% CI: 2.070–143.462, *p* = 0.008), as did those in the respiratory system (OR = 16.267, 95% CI: 1.966–134.567, *p* = 0.010). Mammary tumors had an OR of 20.255 (95% CI: 10.067–40.753, *p* < 0.001), highlighting their aggressive nature. Other sites, such as the reproductive system (OR = 3.362, 95% CI: 0.904–12.511, *p* = 0.070) and the oral cavity (OR = 2.208, 95% CI: 1.121–4.346, *p* = 0.022), showed varying degrees of risk.

Age was also a significant predictor of malignancy. Cats over 11 years had the highest risk of malignancy (OR = 4.702, 95% CI: 2.690–8.219, *p* < 0.001) compared to cats under 6 years of age. Cats in the 9–11 year and 6–8 year age groups had ORs of 3.357 (95% CI: 1.985–5.679, *p* < 0.001) and 2.504 (95% CI: 1.518–4.131, *p* < 0.001), respectively, showing an increased risk of malignancy with advancing age.

Sex, however, was not a significant predictor of malignancy. Female cats had an OR of 1.157 (95% CI: 0.774–1.731, *p* = 0.476) compared to male cats, indicating no significant difference in malignancy risk based on sex.

The logistic regression model was well-calibrated, as indicated by the Hosmer–Lemeshow test (*p* = 0.484), and explained a significant proportion of the variance in malignancy (Nagelkerke R^2^ = 0.451).

## 4. Discussion

This study provides valuable epidemiological data on feline tumors in Korea, addressing a significant gap in the existing literature. While numerous studies have examined feline tumors in Western countries, large-scale, region-specific studies in Asia are much fewer. Research on feline tumor prevalence within Asian populations, particularly in Korea, remains limited. This study addresses the gap in data on feline tumors in Korea, a region where large-scale, specific studies are less common compared to Western countries. Our findings are particularly notable for the remarkably low prevalence of squamous cell carcinoma (SCC), which contributes to a broader understanding of regional variations in tumor characteristics across different feline populations.

### 4.1. Age as a Key Predictor of Malignancy

Age was identified as a significant predictor of malignancy in this study. The frequency of malignant tumors increased significantly with advancing age (*p* < 0.001), with the mean age of cats diagnosed with malignant tumors being 9.33 years, compared to 6.60 years for cats diagnosed with benign tumors. These findings are consistent with studies conducted in other regions. For example, the Swiss Feline Cancer Registry reported a higher frequency of malignant tumors in older cats, noting that the likelihood of tumor development increased with age [10]. Similarly, recent data from the United Kingdom showed that malignant tumors predominantly affected older cats, with a mean age of 12 years, further supporting age as a risk factor for malignancy [12]. In contrast, Manuali et al., in their study of domestic shorthairs in Europe, did not observe a significant statistical correlation between age and malignancy for any tumor type [11]. Huber et al. reported a prevalence of malignant tumors across all age groups, but statistical analysis revealed no notable differences [15].

The regression analysis in this study demonstrated a statistically significant increase in the risk of malignancy with advancing age, with the risk divided into quartiles. The risk increased markedly in cats aged 6–8 years (Q2 group) and further in those aged 9–11 years (Q3 group). Notably, cats aged 12 years and older (Q4 group) exhibited the highest risk of malignancy (OR = 4.702, 95% CI: 2.690–8.219, *p* < 0.001), confirming the significantly elevated risk in older cats.

These results align with previous studies indicating that the risk of malignancy increases with age [10,12,16], suggesting a similar pattern in the Korean cat population. This underscores the importance of regular health monitoring and early screening for older cats to facilitate early detection and treatment of malignancies. Moreover, the development of tailored intervention strategies that account for age-specific risks may improve the quality of life for aging cats.

### 4.2. Influence of Sex on Feline Tumors

In our study, 65.0% of malignant tumors occurred in female cats, compared to 40.5% in male cats (*p* < 0.001). However, multiple logistic regression analysis showed that sex was not a significant predictor of tumor malignancy. This may be because mammary tumors, which are mostly malignant, occur almost exclusively in female cats. A similar pattern was observed in the study by Huber et al., where female cats in Croatia had a higher prevalence of malignant tumors, largely due to the high prevalence of mammary gland tumors [15].

Although cutaneous lymphoma cases were relatively few in our study, lymphoma was the most common tumor in the alimentary tract, accounting for 73.3% (n = 22) of tumors in this region. Lymphoma in the alimentary tract was more frequent in male cats (68.2%), supporting the findings from the Swiss Feline Cancer Registry, which reported a higher prevalence of lymphoma in males [25]. Mammary gland tumors, on the other hand, were predominantly seen in females. While sex may not be a strong overall predictor of tumor malignancy, it could still have some influence on the occurrence of certain tumor types.

### 4.3. Breed Related Differences in Feline Tumors

In our study, the most prevalent breed was the Korean Shorthair, which showed a nearly equal distribution of benign and malignant tumors. Persian cats had a higher prevalence of malignant tumors (69.4%), and the Russian Blue also exhibited a notable prevalence of malignancy (63.6%). However, no statistically significant association between breed and tumor malignancy was found (*p* = 0.173). This may be due in part to the lower proportion of purebred cats in our cohort, which could have limited our ability to detect significant breed-related differences.

Interestingly, other studies have suggested that certain breeds may indeed be predisposed to specific tumor types. For example, Miller et al. reported a higher prevalence of mast cell tumors (MCTs) in Siamese cats [13], and the Swiss Feline Cancer Registry found that some breeds had significantly different odds ratios for developing tumors compared to European Shorthairs [10]. Additionally, differences in the development of certain tumor types, such as fibrosarcoma and squamous cell carcinoma, have been observed across breeds [25]. These findings indicate that while our study did not show a statistically significant association, the role of breed in tumor development cannot be ruled out. Further research with larger cohorts and a more balanced representation of purebred cats may help clarify the influence of breed on tumor susceptibility.

### 4.4. Anatomic Location and Tumor Malignancy

This study identified a significant association between the anatomical location of the tumor and the prevalence of specific malignant tumor types commonly found in those regions. Tumors in the mammary gland and alimentary system showed notably high malignancy rates, with malignancy odds reaching 93.1% (OR = 20.255) for mammary tumors and 93.3% (OR = 34.927) for alimentary tract tumors. These findings underscore that these anatomic sites are more likely to develop malignant tumors, such as carcinomas in mammary gland and lymphomas in the alimentary tract, which is consistent with multiple previous studies.

In this cohort, the malignancy rate for mammary tumors was 93.1%, closely aligning with rates reported in other regions: 97.3% in Italy [11], 83% in Croatia [15], 88.5% in Portugal [16], and 83% in Switzerland [10]. These findings emphasize the aggressive nature of mammary tumors in cats and the importance of timely diagnosis and prompt treatment. Similarly, the pronounced malignancy rate observed in the alimentary tract is primarily due to lymphomas. It is generally observed that the slow-progressing small-cell type is more common in this region [24], although our study did not specifically classify lymphomas by subtype.

### 4.5. Prevalence Differences between Squamous Cell Carcinoma (SCC) and Mast Cell Tumor (MCT)

Of the 408 skin and soft tissue tumors, MCTs were the most frequently observed, accounting for 215 cases (52.7%). Among these, 172 were benign, representing approximately 80% of all MCTs. Given that MCTs are often associated with a relatively favorable prognosis, this high proportion of benign classifications is in line with expectations.

In contrast, SCC was notably rare in this study, with only three cases identified (0.7% of skin and soft tissue tumors). This stands in contrast to findings from other studies, where SCC was more prevalent. For example, Manuali et al. reported SCC as the most common malignant skin tumor, representing 28.8% of cases [11]. This discrepancy may be partly explained by the fact that many cats seen in veterinary clinics in Korea are kept predominantly indoors. This lifestyle significantly reduces their exposure to ultraviolet (UV) radiation, a well-known risk factor for cutaneous SCC [26]. However, despite the low occurrence of skin SCC, the prevalence of oral SCC was substantial, representing 39% of oral tumors (16 out of 41 cases). This suggests that risk factors such as chronic inflammation and viral infections, which are not related to UV exposure, continue to drive SCC development even in indoor environments [27].

The high prevalence of MCTs in our study is strikingly higher than the 2.8% to 21% range reported in other studies. One potential explanation for this elevated prevalence may be the visibility of these tumors on the skin, leading to more frequent detection and diagnosis by pet owners. Additionally, the low prevalence of SCC in our cohort may have skewed the overall distribution of skin tumors, making the proportion of MCTs appear disproportionately high. Breed predispositions were examined, but no significant correlation was identified, indicating that other unknown factors may contribute to this high prevalence of MCTs.

### 4.6. Mast Cell Tumor Prognosis and Mammary Carcinoma Grading

In this study, we applied a classification system based on prognostic criteria to distinguish MCTs as benign or malignant, providing a more detailed understanding of tumor behavior in Korean cats. A particularly important finding was the significant association between age and MCT malignancy, as demonstrated by the chi-square test (*p* < 0.001). This suggests that age is a critical factor in determining the likelihood of malignancy, with older cats showing a significantly higher risk of developing malignant MCTs compared to younger cats.

For mammary tumors, a statistically significant relationship was also observed between age quartiles and the benign or malignant status of the tumors (*p* < 0.001). When focusing on malignant cases, the incorporation of recent grading information revealed that approximately half of the malignant mammary tumors were classified as high-grade, consistent with findings from other studies [21,28]. However, no significant association was identified between histological grade and age, indicating that while age influences the likelihood of malignancy, it does not appear to affect the aggressiveness of the tumor once malignancy is established.

### 4.7. Limitations

It is important to acknowledge the limitations of this study. First, as a retrospective study, it is inherently susceptible to selection bias and may not fully account for all relevant variables that influence tumor development and malignancy in the feline population. The reliance on histopathology records constrains the capacity to control for confounding variables and may impact the generalizability of the findings.

Secondly, IHC was not conducted in all diagnostic cases. Consequently, cases with ambiguous histopathological features were excluded from the analysis. This exclusion may have resulted in an underestimation of certain tumor types, which could impact the accuracy of the reported prevalence and malignancy rates.

Third, the dataset demonstrated a higher prevalence of cases involving the skin and mammary regions, with relatively fewer cases from other anatomical sites. This uneven distribution may introduce bias in the overall findings and limit the applicability of the results to tumors in less represented regions.

Finally, while this study noted a remarkably low frequency of SCC, factors that might directly support this observation, such as lifestyle, environmental exposures, and genetic predispositions, were not analyzed. The lack of these data precludes a comprehensive understanding of the underlying causes of the low SCC prevalence.

### 4.8. Insights and Future Directions in Felime Tumor Research

This study employs regression analysis to explore the effects of age, breed, and anatomical location on feline tumor malignancy. Unlike previous studies in Korea [17], our research is based on a more comprehensive dataset, incorporating detailed grading of mammary tumors and prognosis-based classification of MCTs. These advancements are critical for developing more targeted veterinary interventions and represent a meaningful step forward in understanding the complex dynamics of tumor development in cats.

Feline tumors, particularly mammary gland tumors, often display a notably higher proportion of malignant, high-grade cases compared to dogs, as supported by the existing literature [29,30]. This study’s findings align with these reports, highlighting the elevated prevalence of high-grade malignant mammary tumors in cats. Additionally, the higher frequency of malignant tumors in other internal organs further underscores the role of cats as an important model in comparative oncology research.

Future research should expand on this foundation by incorporating larger, multicenter datasets that include detailed environmental, lifestyle, and genetic information. Such an expansion would allow for a more nuanced investigation of the factors influencing tumor development in cats across Korea and potentially other regions. Comparative studies between Korean cats and those from different geographical areas are also recommended to better understand regional variations in tumor behavior and treatment outcomes.

By deepening our understanding of these regional differences, veterinary medicine can advance towards more personalized care, optimizing prevention and treatment strategies tailored to the specific needs of feline populations. This approach will not only enhance the global veterinary knowledge base, but also improve the precision and efficacy of care for feline patients, ultimately leading to better clinical outcomes.

## 5. Conclusions

This study provides critical insights into the patterns of tumor malignancy in Korean cats, highlighting the significant roles of age and anatomical location in predicting tumor behavior. Older cats were found to have a significantly higher risk of developing malignant tumors, particularly in the mammary gland and alimentary tract, where malignancy rates were exceptionally high. These findings align with previous research showing that feline tumors, especially mammary tumors, tend to be more aggressive than those in dogs.

Although breed was not a statistically significant predictor of malignancy in this study, certain breeds, such as Persians and Russian Blues, exhibited a higher frequency of malignant tumors, warranting further investigation. The low prevalence of SCC in the skin and the high prevalence of MCTs were also notable findings, possibly reflecting lifestyle or environmental factors unique to the region.

Moving forward, expanding this research with larger, multicenter datasets and more detailed genetic and environmental information will be essential to deepening our understanding of feline tumor dynamics. Such studies will help refine veterinary practices and improve outcomes through more targeted, evidence-based interventions.

Overall, this study contributes valuable data to the field of veterinary oncology and emphasizes the importance of continued research in comparative oncology, benefiting both feline and human cancer research.

## Figures and Tables

**Table 1:** Distribution of feline tumors by anatomic location.

Site	Diagnosis	Mean SD Age (Years)	Sex (Number of Cases; %)	
			Male	Female
Skin and soft tissue (n = 408)	Mast cell tumor, benign (n = 172)	5.66 ± 3.57	106 (61.6%)	66 (38.4%)
	Mast cell tumor, malignant (n = 43)	8.91 ± 3.64	25 (58.1%)	18 (41.9%)
	Lipoma (n = 51)	8.16 ± 2.78	27 (52.9%)	24 (47.1%)
	Fibroma (n = 10)	4.80 ± 3.33	8 (80.0%)	2 (20.0%)
	Fibrosarcoma (n = 35)	7.51 ± 3.11	11 (31.4%)	24 (68.6%)
	Trichoblastoma (n = 19)	8.21 ± 3.12	15 (78.9%)	4 (21.1%)
	Peripheral nerve sheath tumor (n = 12)	7.25 ± 3.57	5 (41.7%)	7 (58.3%)
	Basal cell carcinoma (n = 11)	10.09 ± 3.96	4 (36.4%)	7 (63.6%)
	Lymphoma (n = 8)	8.63 ± 3.54	5 (62.5%)	3 (37.5%)
	Apocrine adenoma (n = 1)	13.00	0 (0%)	1 (100%)
	Apocrine carcinoma (n = 6)	10.67 ± 4.68	3 (50.0%)	3 (50.0%)
	Melanocytoma (n = 2)	3.50 ± 3.54	1 (50.0%)	1 (50.0%)
	Amelanotic melanoma (n = 4)	5.00 ± 1.41	3 (75.0%)	1 (25.0%)
	Hemangioma (n = 1)	13.00	0 (0%)	1 (100%)
	Hemangiosarcoma (n = 3)	12.00 ± 3.61	2 (66.7%)	1 (33.3%)
	Squamous cell carcinoma (n = 3)	12.67 ± 2.52	2 (66.7%)	1 (33.3%)
	Extraskeletal osteosarcoma (n = 2)	3.50 ± 4.95	1 (50%)	1 (50%)
	Leiomyosarcoma (n = 1)	6.00	0 (0%)	1 (100%)
	Malignant fibrous histiocytoma (n = 1)	1.00	1 (100%)	0 (0%)
	Myxosarcoma (n = 1)	13.00	0 (0%)	1 (100%)
	Plasmactyoma, malignant (n = 1)	8.00	0 (0%)	1 (100%)
	Trichoepithelioma (n = 1)	9.00	0 (0%)	1 (100%)
	Tricholemmoma (n = 1)	3.00	0 (0%)	1 (100%)
Mammary tissue (n = 159)	Mammary carcinoma (n = 148)	10.35 ± 3.03	0 (0%)	148 (100%)
	Mammary adenoma (n = 11)	5.55 ± 4.61	0 (0%)	11 (100%)
Oral cavity (n = 41)	Peripheral odontogenic fibroma (n = 16)	6.31 ± 3.00	8 (50.0%)	8 (50.0%)
	Squamous cell carcinoma (n = 16)	9.31 ± 3.55	6 (37.5%)	10 (62.5%)
	Fibrosarcoma (n = 4)	15.00 ± 2.31	4 (100%)	0 (0%)
	Salivary adenocarcinoma (n = 2)	10.00 ± 2.83	2 (100%)	0 (0%)
	Ameloblastic fibroma (n = 1)	3.00	1 (100%)	0 (0%)
	Extramedullary plasmacytoma (n = 1)	10.00	1 (100%)	0 (0%)
	Mast cell tumor (n = 1)	15.00	0 (0%)	1 (100%)
Alimentary tract (n = 30)	Lymphoma (n = 22)	8.41 ± 4.14	15 (68.2%)	7 (31.8%)
	Intestinal adenocarcinoma (n = 3)	6.33 ± 1.53	1 (33.3%)	2 (66.7%)
	Gastric adenoma (n = 1)	7.00	1 (100%)	0 (0%)
	Gastrointestinal stromal tumor (n = 1)	5.00	0 (0%)	1 (100%)
	Leiomyoma (n = 1)	1.00	1 (100%)	0 (0%)
	Leiomyosarcoma (n = 1)	3.00	0 (0%)	1 (100%)
	Osteosarcoma (n = 1)	7.00	0 (0%)	1 (100%)
Reproductive system (n = 11)	Dysgerminoma (n = 2)	9.00 ± 4.24	0 (0%)	2 (100%)
	Uterine adenocarcinoma (n = 2)	9.00 ± 1.41	0 (0%)	2 (100%)
	Leiomyosarcoma (n = 2)	4.50 ± 2.12	0 (0%)	2 (100%)
	Lipoma (n = 1)	0	0 (0%)	1 (100%)
	Luteoma (n = 1)	0	0 (0%)	1 (100%)
	Uterine adenoma (n = 1)	14.00	0 (0%)	1 (100%)
	Interstitial cell tumor (n = 1)	11.00	0 (0%)	1 (100%)
	Granulosa cell tumor (n = 1)	6.00	0 (0%)	1 (100%)
Urinary system (n = 10)	Renal cell carcinoma (n = 6)	11.00 ± 2.10	4 (66.7%)	2 (33.3%)
	Urothelial carcinoma (n = 3)	8.33 ± 7.57	2 (66.7%)	1 (33.3%)
	Leiomyoma (n = 1)	6.00	1 (100%)	0 (0%)
Respiratory system (n = 9)	Lymphoma (n = 3)	10.33 ± 4.04	2 (66.7%)	1 (33.3%)
	Nasal carcinoma (n = 3)	6.33 ± 2.08	2 (66.7%)	1 (33.3%)
	Pulmonary adenocarcinoma (n = 2)	7.50 ± 0.71	2 (100%)	0 (0%)
	Leiomyoma (n = 1)	6.00	0 (0%)	1 (100%)
Musculoskeletal system (n = 8)	Osteosarcoma (n = 5)	8.00 ± 3.46	4 (80.0%)	1 (20.0%)
	Chondrosarcoma (n = 2)	9.50 ± 3.54	1 (50.0%)	1 (50.0%)
	Rhabdomyosarcoma (n = 1)	6.00	0 (0%)	1 (100%)
Hemolymphatic system (n = 8)	Lymphoma (n = 4)	5.25 ± 2.87	2 (50.0%)	2 (50.0%)
	Extramedullary plasmacytoma (n = 2)	12.50 ± 0.71	1 (50.0%)	1 (50.0%)
	Fibrosarcoma (n = 1)	16.00	0 (0%)	1 (100%)
	Hemangioma (n = 1)	6.00	1 (100%)	0 (0%)
Body cavity, Eye, and Liver (n = 5)	Hepatocellular carcinoma (n = 2)	12.00 ± 2.83	1 (50.0%)	1 (50.0%)
	Iridociliary adenoma (n = 1)	4.00	0 (0%)	1 (100%)
	Mesothelioma (n = 1)	10.00	0 (0%)	1 (100%)
	Thymic carcinoma (n = 1)	11.00	0 (0%)	1 (100%)

**Table 2:** Logistic regression analysis of tumor malignancy based on location, sex, and age in feline cases.

Category		*p* Value	Exp(B)	95% CI for Exp(B)	
				Lower	Upper
Location	Skin and subcutis (n = 408)	Reference			
	Mammary tissue (n = 159)	<0.001	20.255	10.067	40.753
	Oral cavity (n = 41)	0.022	2.208	1.121	4.346
	Alimentary tract (n = 30)	<0.001	34.927	7.998	152.521
	Reproductive system (n = 11)	0.070	3.362	0.904	12.511
	Urinary system (n = 10)	0.008	17.234	2.070	143.462
	Respiratory system (n = 9)	0.010	16.267	1.966	134.567
	Others (n = 21)	<0.001	19.411	4.326	87.090
Sex	Male (n = 306)	Reference			
	Female (n = 383)	0.476	1.157	0.774	1.731
Age	<6 years (n = 200)	Reference			
	6–8 years (n = 175)	<0.001	2.504	1.518	4.131
	9–11 years (n = 169)	<0.001	3.357	1.985	5.679
	>11 years (n = 145)	<0.001	4.702	2.690	8.219

## Data Availability

The original contributions presented in this study are included in the article/Supplementary Materials, and further inquiries can be directed to the corresponding author/s.

## Supplementary Materials

The following supporting information can be downloaded at: https://www.mdpi.com/article/10.3390/ani14202989/s1, Table S1: Clinical information of the cohort.
